# Effect of Opt-In vs Opt-Out Framing on Enrollment in a COVID-19 Surveillance Testing Program: The COVID SAFE Randomized Clinical Trial

**DOI:** 10.1001/jamanetworkopen.2021.12434

**Published:** 2021-06-03

**Authors:** Allison H. Oakes, Jonathan A. Epstein, Arupa Ganguly, Sae-Hwan Park, Chalanda N. Evans, Mitesh S. Patel

**Affiliations:** 1 VA Health Services Research & Development Center for Health Equity Research and Promotion, Crescenz Veterans Affairs Medical Center, Philadelphia, Pennsylvania; 2 Penn Medicine Nudge Unit, University of Pennsylvania, Philadelphia; 3 Perelman School of Medicine, University of Pennsylvania, Philadelphia; 4 Genetic Diagnostic Laboratory, Department of Genetics, University of Pennsylvania, Philadelphia; 5 Wharton School, University of Pennsylvania, Philadelphia

## Abstract

This randomized clinical trial examines the effect of an opt-out recruitment strategy vs a conventional opt-in strategy on enrollment and initial adherence to a COVID-19 surveillance testing program.

## Introduction

The SARS-CoV-2 (COVID-19) pandemic has caused workplaces and campuses to close or shift many people to remote work. To safely reopen, surveillance testing is needed to help to identify asymptomatic and presymptomatic cases.^1,2^ We conducted a clinical trial to rapidly implement and scale a saliva-based method for COVID-19 surveillance testing.^3^ However, participation in clinical trials is often suboptimal. In prior work,^4,5^ opt-out framed recruitment strategies have shown promise for increasing program enrollment; this approach may leverage status quo bias. In this randomized clinical trial, we tested the effect of an opt-out framed recruitment strategy compared with a conventional opt-in strategy on enrollment and initial adherence to a COVID-19 testing program.

## Methods

The COVID-19 Screening Assessment for Exposure Trial (COVID SAFE) was a randomized clinical trial conducted between September 9, 2020, and October 30, 2020 (ClinicalTrials.gov identifier NCT04506268). The trial protocol (Supplement 1) was approved by the University of Pennsylvania institutional review board. This study followed the Consolidated Standards of Reporting Trials (CONSORT) reporting guideline.

On the basis of power calculations informed by the existing literature, participants were electronically randomized into opt-out and opt-in groups using a 1:2 allocation ratio.^4,5^ Eligible participants included faculty, staff, and students at the University of Pennsylvania who were aged 18 years or older, on campus at least 1 day per week, and owned a smartphone. Recruitment emails were sent from the Office of the Executive Vice Dean. Those in the opt-in group were emailed an invitation to enroll and given a link to get more information, whereas those in the opt-out group were told they were conditionally enrolled and given a link to complete the process. The study was conducted using Way to Health, a research platform at the University of Pennsylvania. Interested participants accessed the study website to create an account, provide informed consent, and complete surveys. Participants were asked to complete a biweekly saliva-based COVID-19 screening test for up to 6 months.

The primary outcome was the proportion of participants who enrolled within 4 weeks of invitation. The secondary outcome was the proportion of participants who completed their first scheduled screening test. We fit models for the outcome measures according to generalized estimating equations with a logit link and an exchangeable correlation structure using participant as the clustering unit.

The model included participant age, sex, race/ethnicity, and date of invitation. Race/ethnicity was assessed because enrollment in clinical trials is suboptimal, and the characteristics of enrolled individuals are often not representative of the general population. Recent work^5^ suggests that opt-out framing might help address this issue. At the point of invitation, race/ethnicity data came from administrative employment records. The categories were determined on the basis of a combination of prior work and the distribution of data.

To obtain the adjusted difference and 95% CIs between groups, we used the bootstrap method, resampling participants 2000 times. Investigators and data analysts were blinded to group assignments until the analysis was completed. Two-sided hypothesis tests used an α of .05. Analyses were conducted using the Python statsmodel module version 0.12.1 (Python). Data analysis was conducted from October to December 2020.

## Results

A total of 1759 participants were randomized (eFigure in Supplement 2). Baseline characteristics were similar among groups, including age (mean [SD] age, invited opt-in group, 40.2 [13.3] years; invited opt-out group, 40.0 [13.0] years; enrolled opt-in group, 38.1 [13.3] years; and enrolled opt-out group, 38.8 [12.9] years), sex (invited opt-in group, 566 men [50.4%]; invited opt-out group, 268 men [47.7%]; enrolled opt-in group, 107 men [41.8%]; enrolled opt-out group, 75 men [48.1%]), and race/ethnicity (invited opt-in group, 581 White participants [49.5%]; invited opt-out group, 253 White participants [43.2%]; enrolled opt-in group, 161 White participants [62.9%]; and enrolled opt-out group, 89 White participants [57.1%]) (Table 1). Between study groups, enrolled participants also did not differ in terms of self-reported income or education (Table 1).

**Table 1.** Participant Characteristics

Characteristic	Participants, No. (%)					
	Invited			Enrolled		
	Opt-in (n = 1173)	Opt-out (n = 586)	*P* value	Opt-in (n = 256)	Opt-out (n = 156)	*P* value
Age, mean (SD), y	40.2 (13.3)	40.0 (13.0)	.67	38.1 (13.3)	38.8 (12.9)	.59
Sex						
Male	566 (50.4)	268 (47.7)	.31	107 (41.8)	75 (48.1)	.25
Female	557 (49.6)	294 (52.3)		149 (58.2)	81 (51.9)	
Race/ethnicity						
Non-Hispanic			.05			.75
White	581 (49.5)	253 (43.2)		161 (62.9)	89 (57.1)	
Black	146 (12.4)	78 (13.3)		9 (3.5)	7 (4.5)	
Asian	282 (24.0)	158 (27.0)		47 (18.4)	35 (22.4)	
Hispanic	34 (2.9)	12 (2.0)		10 (3.9)	5 (3.2)	
Multiple or other races/ethnicities^a^	130 (11.1)	85 (14.5)		29 (11.3)	20 (12.8)	
Annual household income, $						
<50 000	NA	NA	NA	63 (24.6)	41 (26.3)	.93
50 000-100 000	NA	NA		74 (28.9)	44 (28.2)	
>100 000	NA	NA		119 (46.5)	71 (45.5)	
Education						
Some high school or less	NA	NA	NA	2 (0.8)	0 (0.0)	.45
Some college or specialized training	NA	NA		7 (2.7)	6 (3.8)	
College graduate or higher	NA	NA		247 (96.5)	150 (96.2)	

Abbreviation: NA, not applicable.

^a^ Other includes Native Hawaiian or other Pacific Islander, American Indian, or Alaska Native.

The opt-out group had significantly greater enrollment than the opt-in group (26.6% [156 of 586] vs 21.8% [256 of 1173 ]; adjusted difference, 5.1 percentage points; 95% CI, 1.0 to 9.3 percentage points; *P* = .01) (Table 2). Among enrolled participants, there was no difference in first test completion (−2.1 percentage points; 95% CI, −8.9 to 4.2 percentage points; *P* = .54), but across the total sample the opt-out group had significantly greater first test completion (3.9 percentage points; 95% CI, −0.0 to 8.1 percentage points; *P* = .04).

**Table 2.** Trial Outcomes

Outcome measures	Participants, No. (%)		Adjusted difference vs opt-in, % (95% CI)	*P* value
	Opt-in (n = 1173)	Opt-out (n = 586)		
Trial enrollment	256 (21.8)	156 (26.6)	5.1 (1.0 to 9.3)	.01
Completion of first test				
Conditioned on enrollment	224 (87.5)	133 (85.3)	−2.1 (−8.9 to 4.2)	.54
Total	224 (19.1)	133 (22.7)	3.9 (0.0 to 8.1)	.04

## Discussion

In this randomized clinical trial, an opt-out framed recruitment strategy increased enrollment into a COVID-19 screening program and increased the overall rate of test completion. This study is limited to a single academic health system. If applied more broadly, the increase of 5.1 percentage points may have substantial implications for uptake. This study is one of the first to examine the effect of default options on enrollment in a COVID-19–related program. These findings could inform other health promotion efforts needed to address the COVID-19 pandemic.^6^

## Notes

Supplement 1. Trial Protocol

Supplement 2. **eFigure. **CONSORT Diagram

Supplement 3. Data Sharing Statement
