# Supplementary material for: Effect of Opt-In vs Opt-Out Framing on Enrollment in a COVID-19 Surveillance Testing Program: The COVID SAFE Randomized Clinical Trial
Source: JAMA Netw Open. 2021 Jun 3;4(6):e2112434. doi: 10.1001/jamanetworkopen.2021.12434 (PMC8176333; doi:10.1001/jamanetworkopen.2021.12434)
Supplement: Supplement 1. — Trial Protocol [file jamanetwopen-e2112434-s001.pdf]

COVID SAFE

COVID-19 Screening Assessment for Exposure

Study Protocol

November 2020

Outline

1. Abstract

2. Overall objectives

3. Aims

    3.1 Primary outcome

    3.2 Secondary outcomes

4. Background

5. Study design

    5.1 Design

    5.2 Study duration

    5.3 Target population

    5.4 Accrual

    5.5 Key inclusion criteria

    5.6 Key exclusion criteria

6. Subject recruitment

7. Subject compensation

8. Study procedures

    8.1 Consent

    8.2 Procedures

9. Analysis plan

10. Investigators

11. Human research protection

    11.1 Data confidentiality

    11.2 Subject confidentiality

    11.3 Subject privacy

11.4 Data disclosure

11.5 Data safety and monitoring

11.6 Risk/benefit

11.6.1 Potential study risks

11.6.2 Potential study benefits

11.6.3 Risk/benefit assessment

12. Bibliography

1. Abstract

In order to safely and effectively reopen businesses and universities across the US, institutions will need to develop approaches to rapidly identify COVID-19 cases and manage their spread while balancing program effectiveness, feasibility, costs, and scalability. We will evaluate the implementation of a COVID-19 screening program that coordinates several existing systems at the University of Pennsylvania including saliva-based viral testing. We will pilot this program to a small cohort of the Penn Medicine workforce and then test different approaches to improve program enrollment that utilize opt-in vs. opt-out framing.

2. Overall objectives

The objective of the study is:

To evaluate the implementation of a COVID-19 screening program that uses saliva-based testing and to determine the effect of an opt-out versus an opt-in approach on program enrollment.

3. Aims

3.1 Primary outcome

The percent of those invited who enroll in the program.

3.2 Secondary outcomes

The distribution of participant sociodemographic characteristics (e.g. age, gender, race/ethnicity), the rate of compliance with saliva-based testing.

3.3 Exploratory outcomes

The cost of the program, barriers and facilitators to scalability, and overall program feasibility.

4. Background

The coronavirus disease 2019 (COVID-19) pandemic has resulted in close to 10,000,000 reported cases worldwide, including more than 2,000,000 aggregated reported cases and 120,000 deaths in the United States [1,2]. Initial efforts to address the COVID-19 pandemic were aimed at testing symptomatic individuals, implementing stay-in-place orders, and at increasing hospital capacity to meet surge demands [3]. While we continue to confront the current crisis, we must plan for the future by putting in place tools to enhance our ability to conduct effective screening, containment, and case management.

Widespread COVID-19 testing is needed to safely and effectively reopen schools and businesses across the US. However, currently approved testing options require reagents that are limited in supply, severely hindering scalability [4]. Emerging evidence indicates that saliva testing with

the option of at-home sample collection can accurately identify COVID-19 viral infection [5]. Additional diagnostic testing options will continue to increase patient access. Moreover, this approach provides an option for the easy, safe and convenient collection of samples required for testing without traveling to a doctor's office, hospital, or testing site. Collection by the patient also reduces exposure of health care workers to the virus and preserves limited personal protective equipment [6].

With access to expanded testing, health systems and universities will need to test alternative methods to manage COVID-19 spread while balancing program effectiveness, feasibility, costs, and scalability. Insights from the field of behavioral economics offer promise for designing and sustaining these kinds of policies. Specifically, research has demonstrated that an opt-out framed recruitment strategy compared to a conventional opt-in strategy can improve enrollment and adherence to behavioral interventions [7-10].

For these reasons, we propose to evaluate the implementation of a COVID-19 screening program that uses saliva-based testing and to test approaches to improve program enrollment.

5. Study design

5.1 Design

We will conduct a clinical trial of at least 6 months beginning with approximately 2500 Penn Medicine employees (faculty, staff and trainees) and potentially increasing as the program rolls out more broadly. In the first phase, we will pilot the approach with up to 50 participants to help optimize implementation. In Phase 2, participants will be randomly assigned to receive an opt-in or opt-out framed study recruitment email. Participants randomly assigned to the opt-in arm will be instructed to visit the Way to Health platform to enroll in the study to call a study coordinator. Participants randomized to the opt-out arm will be told that they have been conditionally enrolled in the voluntary research program and will be instructed to visit the Way to Health platform to get started; participants in this group will receive escalating reminders (e.g. reminder emails, phone call) unless they decline to participate.. Across study arms, interested employees will visit the study website to learn more about the study, create an account, complete a screening survey, provide informed consent, and complete an initial demographic and screening survey.

Enrolled participants will complete additional surveys. All enrolled individuals will be asked to complete and drop off a saliva sample for COVID-19 testing every week (Figure 1). This testing frequency interval may change based on public health and university guidance. Individuals with a positive saliva test will be asked to complete a confirmatory test that is approved by the Clinical Laboratory Improvement Amendments (CLIA) and will be encouraged to self-quarantine while they await results. Individuals with a positive confirmatory test result will be connected to occupational medicine for next steps and will need to follow department specific guidelines for returning to work.

Figure 1. Study Process

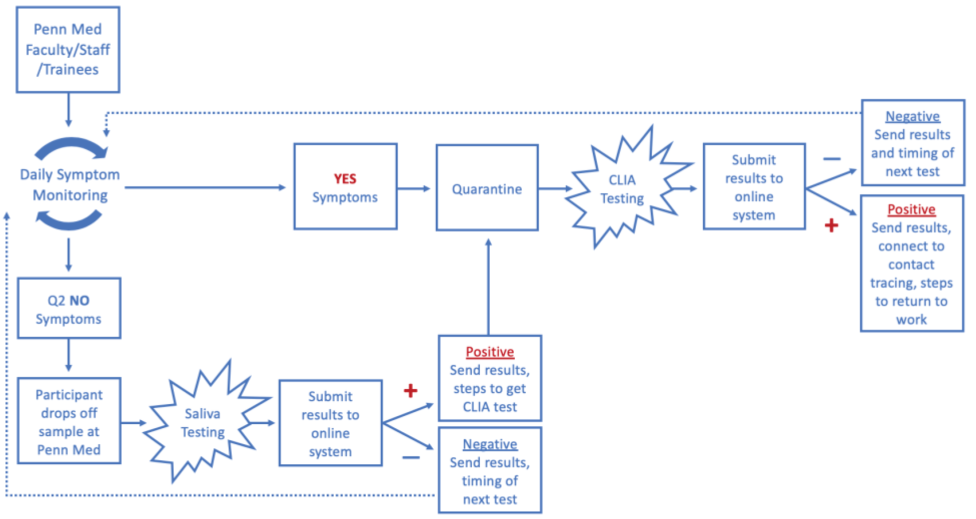

5.2 Study duration

The study is expected to begin in July 2020 and participants will continue in the study for 6 months.

5.3 Target population

The study sample will be drawn from University of Pennsylvania staff, faculty, and trainees that have returned to work on campus who have been identified by leadership at the Perelman School of Medicine.

5.4 Accrual

Potentially eligible employees will be contacted by email and phone to participate in the study and enrolled via the Way to Health Platform.

5.5 Key inclusion criteria

To be eligible patients must:

1) Be 18 years or older
2) Be University of Pennsylvania staff, faculty or trainee
3) Have a phone or device capable of receiving text messages
4) Willing to participate in the study for 6 months

5.6 Key exclusion criteria

Participants will not be eligible if they identify any reason they are unable to participate in the study.

6. Subject recruitment

Leadership from the Perelman School of Medicine will provide an initial list of potentially eligible participants who are University of Pennsylvania faculty, staff, or trainees that have returned to campus for work. We will recruit this population through a combination of emails and phone calls.

7. Subject compensation

No compensation will be offered for this study.

8. Study procedures

8.1 Consent

Informed consent will be obtained electronically through the Way to Health study website since these participants will not be seen in-person. Participants will be able to read through the informed consent and a member of the research team will be available for questions. Once signed, participants will be able to access and print a copy of the combined informed consent/ HIPAA form from the Way to Health online platform. Participants will be instructed that they may reach out to the study team with questions and that, since participation is voluntary, they are able to drop out of the study at any time, and that the decision to enroll or not to enroll will have no impact on their employment status at Penn. Enrollment in the study will be defined as a 4 week window after invitation to join.

8.2 Procedures

Participants will be enrolled in the study using the Way to Health platform and asked to complete an initial screening and demographic questionnaire to assess eligibility. If eligible, participants will then complete an informed consent and HIPAA authorization form. Participants will pick up their test kits and drop off their samples at a designated Penn Medicine location. Participants will be instructed to not eat or drink within 30 minutes of providing their saliva sample at the lab. They will provide a sample of at least 1mL into a 50mL test tube and drop off the sample with designated lab staff. Data on symptoms will be obtained from the mandatory Penn Medicine symptom monitoring program (PennOpen Pass). If a participant remains symptom free, they will complete a saliva-based screening test. Results will be shared with the study team using LabVantage, a Laboratory Information Management System, and the study team will communicate these results to the study participant. If the results of the saliva screening test are negative, data will continue to be collected on symptoms and timing of the next saliva test will be given.

If results of the saliva screening test are positive, participants will be referred to the PennOpen Pass Call Center where they will receive guidance on how to obtain a CLIA approved test to confirm diagnosis of COVID-19. They may also be directed to contact tracing, advised to enter self-quarantine. Upon receipt of a positive result, they will be given next steps for returning to work. If results of the CLIA approved test are negative, participants will re-enter daily symptom monitoring and timing of the next saliva test will be given.

The COVID-19 screening test was developed by University of Pennsylvania faculty and staff on the Rapid Assay Task Force. The task force was assembled under the direction of Dr. Jon Epstein, Chief Scientific Officer and Vice Dean at the Perelman School of Medicine and is led by Dr. Frederic Bushman, Professor and Chair of the Department of Microbiology. The saliva-based test is conducted using a highly sensitive reverse-transcription loop-mediated isothermal amplification (RT-LMAP) assay compatible with reagents developed at the University of Pennsylvania to detect SARS-CoV-2 viral RNA. LAMP is a method of isothermal DNA replication that utilizes, in an accelerated format, DNA oligos that hybridize with different regions of the target molecule. Utilizing a strand displacing polymerase and loops formed during this reaction, a fast amplification reaction can occur upon proper oligo binding to the desired target. Such reactions are capable of generating microgram quantitates of DNA in a very short period of time at a single reaction temperature. Furthermore, although the strand-displacing polymerase has reverse transcriptase activity, a reverse transcriptase can be included to improve sensitivity within the reaction when detecting an RNA target (RT-LAMP). The saliva test developed by the University of Pennsylvania uses SARS-CoV-2 specific primer sets, designed uniquely to detect SARS-CoV-2 RNA. The protocol is simple, inexpensive, and rapid. Results are typically available in about one hour. Positive samples are called by comparison to positive and negative controls; reactions displaying a color shift indicate that the target sequence is present. Positive results are indicative of the presence of SARS-CoV-2-RNA but must be confirmed by a CLIA approved test. Existing reports suggest that assays based upon these protocols allow for a sensitivity down to at least 1 genome per microliter when starting with 0.5 ml of saliva [11].

Quality control (QC) checks will occur at multiple points in this protocol:

1. Sample QC – Verify all samples are contained within the appropriate container, are labeled with two unique identifiers, and that samples have been stored appropriately prior to arrival in the laboratory.

2. Verify that all required materials are available in the necessary quantities prior to protocol initiation.

3. Ensure that all reagents used are within the expiration date and that all reagents used have been properly validated.

4. Prior to starting the procedure, ensure all required tubes have been properly labeled.

5. A positive control QC step, consisting of LAMP reactions spiked with synthetic SARS-CoV-2 RNA, is performed to ensure that the LAMP reaction worked as expected.

6. A negative control QC step, consisting of LAMP reactions spiked with human total RNA, is performed to ensure that no contamination of LAMP reagents has occurred.

9. Analysis plan

We will use an intention-to-treat approach to evaluate the two-arm randomized trial of opt-in vs opt-out framing for recruitment. We will conduct chi-squared tests to compare the proportion of participants that were invited who enrolled in the program. Using a 2:1 allocation favoring the opt-in group, we estimate that about 1200 employees will need to be enrolled to have >90% power to detect a 10 percentage point difference between an enrollment rate of 30% in the opt-in arm and 40% in the opt-out arm. This assumes a 2-sided α of <0.05 as statistically significant.

To evaluate the impact of opt-in vs opt-out recruitment on the population enrolled, we will compare age, gender, race/ethnicity of participants that enrolled in the program.

To evaluate the feasibility of the program, summary statistics will be used among the observational cohort to evaluate trends of compliance with saliva-based testing, barriers and facilitators to implementation, and costs of the program.

10. Investigators

Mitesh Patel, MD, MBA is the Principal Investigator (PI) and is the Ralph Muller Presidential Associate Professor of Medicine and Health Care Management at the Perelman School of Medicine and The Wharton School at the University of Pennsylvania. He is the Director of the Penn Medicine Nudge Unit and has led more than 25 randomized clinical trials including many behavioral interventions that use the Way to Health research information technology platform. He has experience and training in behavioral economics, clinical trial design and analysis, health services research, and statistical analysis.

11. Human research protection

11.1 Data confidentiality

Paper-based records will be kept in a secure location and only be accessible to personnel involved in the study. Computer-based files will only be made available to personnel involved in the study through the use of access privileges and passwords. Wherever feasible, identifiers will be removed from study-related information. Lab-based data and test kits will use a barcode linked to the participant study number. In the event of a positive test result, we will use this information to notify the participant. Lab staff may be privy to participant identity but have been trained to maintain confidentiality. Precautions are in place to ensure the data are secure by using passwords and encryption, because the research involves web-based surveys.

11.2 Subject confidentiality

Research material will be obtained from participant surveys and viral testing. All participants will provide informed consent for access to these materials. The data to be collected include data on participant daily symptoms and outcomes of viral testing. Research material that is obtained will be used for research purposes only. The same procedure used for the analysis of automated data sources to ensure protection of patient information will be used for the survey data, in that patient identifiers will be used only for linkage purposes or to contact patients. The study identification number, and no other identifying information, will be used on all data collection instruments. All study staff will be reminded to appreciate the confidential nature of the data collected and contained in these databases. The Penn Medicine Academic Computing Services (PMACS) will be the hub for the hardware and database infrastructure that will support the project and is where the Way to Health web portal is based. The PMACS is a joint effort of the University of Pennsylvania's Abramson Cancer Center, the Cardiovascular Institute, the Department of Pathology, and the Leonard Davis Institute. The PMACS provides a secure computing environment for a large volume of highly sensitive data, including clinical, genetic, socioeconomic, and financial information. Among the IT projects currently managed by PMACS are: (1) the capture and organization of complex, longitudinal clinical data via web and clinical applications portals from cancer patients enrolled in clinical trials; (2) the integration of genetic array databases and clinical data obtained from patients with cardiovascular disease; (3) computational biology and cytometry database management and analyses; (4) economic and health policy research using Medicare claims from over 40 million Medicare beneficiaries. PMACS requires all users of data or applications on PMACS servers to complete a PMACS-hosted cybersecurity awareness course annually, which stresses federal data security policies under data use agreements with the university. The curriculum includes Health Insurance Portability and Accountability Act (HIPAA) training and covers secure data transfer, passwords, computer security habits and knowledge of what constitutes misuse or inappropriate use of the server. We will implement multiple, redundant protective measures to guarantee the privacy and security of the participant data. All investigators and research staff with direct access to the identifiable data will be required to undergo annual responsible conduct of research, cybersecurity, and HIPAA certification in accordance with University of Pennsylvania regulations.

Data will be stored, managed, and analyzed on a secure, encrypted server behind the University of Pennsylvania Health System (UPHS) firewall. This server was created for projects conducted by the Penn Medicine Nudge Unit related to physician and patient behavior at UPHS. All study personnel that will use this data are listed on the IRB application and have completed training in HIPAA standards and the CITI human subjects research. Data access will be password protected. Whenever possible, data will be de-identified for analysis.

11.3 Subject privacy

Interested participants will be directed to the Way to Health portal where they will be asked to enter data related to eligibility and their demographic characteristics. Enrollment will include a description of the voluntary nature of participation, the study procedures, risks and potential benefits in detail. The enrollment procedure will provide the opportunity for potential participants to ask questions and review the consent form information with family and friends prior to making a decision to participate. Participants will be told that they do not have to answer any questions if they do not wish and can drop out of the study at any time, without affecting their medical care or the cost of their care. They will be told that they may or may not benefit directly from the study and that all information will be kept strictly confidential, except as required by law. Subjects will have access to a copy of the consent document.. There is the possibility that a participant's involvement in the study may be made known to other participants, as other study participants may be visiting the lab at the same time. We will do our best to stagger sample drop off days to avoid contact with others. Other study participants will not have access to any personal information or other information collected throughout this study. All efforts will be made by study staff to ensure subject privacy.

11.4 Data disclosure

The following entities, besides the members of the research team, may receive protected health information (PHI) for this research study: Penn Medicine, the health system where essential employees enrolled in the study are employed. The Office of Human Research Protections at the University of Pennsylvania -Federal and state agencies (for example, the Department of Health and Human Services, the National Institutes of Health, and/or the Office for Human Research Protections), or other domestic or foreign government bodies if required by law and/or necessary for oversight purposes.

11.5 Data safety and monitoring

The Principal Investigator and research team will closely monitor the safety, privacy, and data integrity of the study. Patients will be provided contact information for study staff and if adverse events are identified, events will be reported and brought to the PI's attention.

11.6 Risk/benefit

11.6.1 Potential study risks

All data described previously will be protected as described in the Subject confidentiality section. There is minimal risk to subjects as there is minimal risk of breach of data. Our team has extensive experience working with these types of data. We will use commercial-grade encryption to protect participant information. Their personal information will only be used by study team members who have been trained to use secure protocols to maintain the privacy of data. Risks and side effects related to this study include the possibility that answering certain questions on the surveys about symptoms may make study participants feel slightly uncomfortable or may be

inconvenient. The risks of providing a saliva sample for testing may include the possibility for false positives or false negatives, testing may induce feelings of fear or anxiety, a positive test result may cause participants to miss days of work, and individuals may experience mild discomfort if they receive the CLIA approved test. We expect the risks of picking up and dropping of test kits to be no more than the risk of returning to campus.

11.6.2 Potential study benefits

This unique program could improve the health of individuals by picking up on changes in symptoms earlier allowing COVID positive participants to be identified quickly and reduce the spread of the virus. However, participants may receive no benefit from the study.

11.6.3 Risk/benefit assessment

There is minimal risk of breach of data and appropriate measures have been taken. Therefore, we believe the risk/benefit assessment is favorable given the potential insights that could be yielded from the findings of this study.

12. References

1. Stokes EK, Zambrano LD, Anderson KN, et al. Coronavirus Disease 2019 Case Surveillance—United States, January 22 – May 30, 2020. MMWR Morb Moral Wkly Rep 2020;69:759-765.
2. Dong E, Du H, Gardner L. An interactive web-based dashboard to track COVID-19 in real time. Lancet Infect Dis; published online Feb 19.
3. McClellan M, Gottlieb S, Mostashari F, et al. A national COVID-19 surveillance system: Achieving containment. Accessed June 29, 2020. https://healthpolicy.duke.edu/sites/default/files/atoms/files/covid-19_surveillance_roadmap_final.pdf
4. Association of State and Territorial Health Officials, Association of Public Health Laboratories, and Council of State and Territorial Epidemiologists. COVID-19 Testing Needs to Be Limited to Priority Groups Unit Sufficient Testing Supplies and Personal Protective Equipment is Available Nationwide. Accessed June 29, 2020. https://www.astho.org/Press-Room/COVID-19-Testing-Needs-to-Be-Limited-to-Priority-Groups-for-Now/03-20-20/
5. U.S. Food & Drug Administration. Coronavirus (COVID-19) Update: FDA Authorizes First Diagnostic Test Using At-Home Collection of Saliva Specimens. Accessed June 29, 2020. https://www.fda.gov/news-events/press-announcements/coronavirus-covid-19-update-fda-authorizes-first-diagnostic-test-using-home-collection-saliva
6. Tu YP, Jennings R, Cangelosi GA, et al. Swabs collected by patients or health care workers for SARS-CoV-2 Testing. N Engl J Med. 2020. PMID: 32492294.
7. Aysola J, Tahirovic E, Troxel AB, et al. A randomized controlled trial of opt-in versus opt-out enrollment into a diabetes behavioral intervention. Am J health Promot. 2018;32:745-752.
8. Mehta SJ, Khan T, Guerra C, et al. A randomized controlled trial of opt-in versus opt-out colorectal cancer screening outreach. Am J Gastroenterol. 2018;113:1848-1854.

9. Mehta SJ, Feingold J, Vandertuyn M, et al. Active choice and financial incentives to increase rates of screening colonoscopy—A randomized controlled trial. Gastroenterology. 2017;153:1227-1229.e2.
10. Mehta SJ, Troxel AB, Marcus N, et al. Participation rates with opt-out enrollment in a remote monitoring intervention for patients with myocardial infarction. JAMA Cardiol. 2016;1:847-848.
11. Rabe BA, Cepko C. SARS-CoV-2 detection using an isothermal amplification reaction and a rapid, inexpensive protocol for sample inactivation and purification. medRxiv preprint doi: https://doi.org/10.1101/2020.04.23.20076877. Accessed July 7, 2020.
