# Supplementary material for: Effect of Opt-In vs Opt-Out Framing on Enrollment in a COVID-19 Surveillance Testing Program: The COVID SAFE Randomized Clinical Trial
Source: JAMA Netw Open. 2021 Jun 3;4(6):e2112434. doi: 10.1001/jamanetworkopen.2021.12434 (PMC8176333; doi:10.1001/jamanetworkopen.2021.12434)
Supplement: Supplement 2. — eFigure. CONSORT Diagram [file jamanetwopen-e2112434-s002.pdf]

Supplemental Online Content

Oakes AH, Epstein JA, Ganguly A, Park SH, Evans CN, Patel MS. Effect of opt-in vs opt-out framing on enrollment in a COVID-19 surveillance testing program: the COVID SAFE randomized clinical trial. *JAMA Netw Open*. 2021;4(6):e2112434. doi:10.1001/jamanetworkopen.2021.12434

eFigure. CONSORT Diagram

This supplemental material has been provided by the authors to give readers additional information about their work.

© 2021 Oakes AH et al. *JAMA Network Open.*

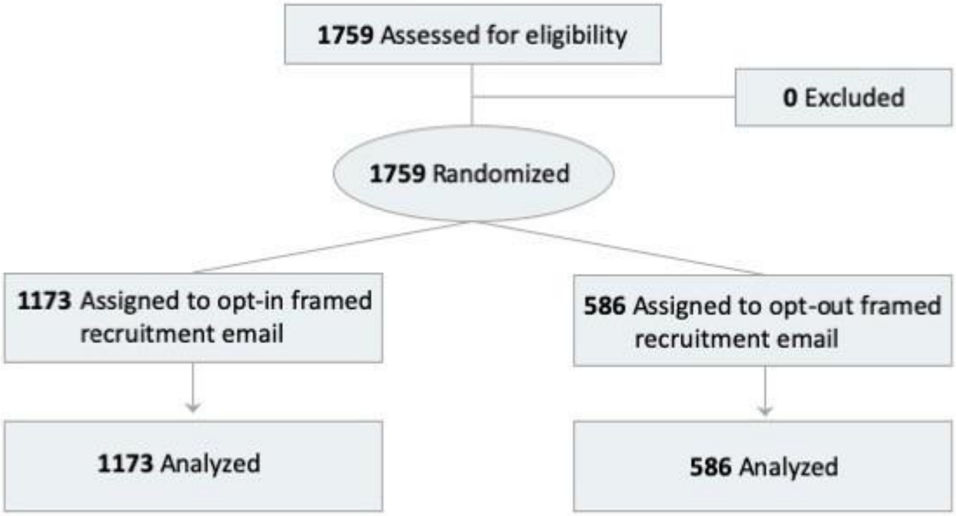

eFigure. CONSORT Diagram Participants were randomly assigned to one of two arms.

© 2021 Oakes AH et al. *JAMA Network Open.*
